# 

**Published:** 1855-07

**Authors:** 


### EDITORIAL.

*To our Subscribers:*

When the business department of the Journal passed fully into the hands of the undersigned, at the commencement of the present volume, he promised to make its publication *regular* on the first of every month, and, provided the subscribers would pay up promptly, to improve its whole mechanical and typographical appearance. The first of these promises he has fathfully redeemed, the Journal having been published and mailed regularly during the first few days of each month; and with the present number he commences a very marked improvement in other respects.— The subscribers, however, have hardly done their part. For though more than half of the present volume is now published, yet not *one-third* of those whose names stand on our subscription book have paid for the present year; and there are two or three hundred who have not been credited a single dime during the last *three years.* Such a condition of things ought never to exist. It is unjust to all parties concerned, and is generally the result of simple individual carelessness and procrastination. Two dollars a year is a very small sum, and, of itself, not worth the trouble of collection; but *two* dollars due from each of 1000 or 1100 subscribers, constitutes a sum of very great importance to the publisher of a Journal who is paying the money every month for paper, printing, binding, &c., &c. It is hoped that this simple statement will be sufficient to cause every subscriber who has not paid already, to send along the money immediately.

Bills will be sent to some of those indebted to us, in the present number of the Journal, and to the remaining ones in our next issue. If any receive *bills* that they consider incorrect, they will confer a favor by informing the undersigned without delay, and all errors will be promptly corrected. It will be remembered that those who are in arrears for past volumes, are liable for $3.00 per annum.

All letters should be directed to N. S. DAVIS, Chicago, Ill.

